# scSemiAE: a deep model with semi-supervised learning for single-cell transcriptomics

**DOI:** 10.1186/s12859-022-04703-0

**Published:** 2022-05-05

**Authors:** Jiayi Dong, Yin Zhang, Fei Wang

**Affiliations:** 1 Shanghai Key Lab of Intelligent Information Processing, Fudan University, Shanghai, China; 2 School of Computer Science and Technology, Fudan University, Shanghai, China

**Keywords:** Semi-supervised, Dimensionality reduction, Autoencoder, Fine-tuning

## Abstract

**Background:**

With the development of modern sequencing technology, hundreds of thousands of single-cell RNA-sequencing (scRNA-seq) profiles allow to explore the heterogeneity in the cell level, but it faces the challenges of high dimensions and high sparsity. Dimensionality reduction is essential for downstream analysis, such as clustering to identify cell subpopulations. Usually, dimensionality reduction follows unsupervised approach.

**Results:**

In this paper, we introduce a semi-supervised dimensionality reduction method named scSemiAE, which is based on an autoencoder model. It transfers the information contained in available datasets with cell subpopulation labels to guide the search of better low-dimensional representations, which can ease further analysis.

**Conclusions:**

Experiments on five public datasets show that, scSemiAE outperforms both unsupervised and semi-supervised baselines whether the transferred information embodied in the number of labeled cells and labeled cell subpopulations is much or less.

**Supplementary Information:**

The online version contains supplementary material available at 10.1186/s12859-022-04703-0.

## Background

In order to grasp the heterogeneous information of cells and cut costs of material and time, Tang et al. [1] first proposed the single-cell RNA sequencing (scRNA-seq) technology in 2009 which can describe the transcriptional profiles from the perspective of a single cell rather than the average of all cells. With the rapid development in recent years, modern single-cell sequencing platforms such as SmartSeq [2] and Chromium 10X [3] have emerged and the number of gene expression profiles has surged to hundreds of thousands [4], which has aroused much interest of researchers. Through analyzing these data, not only can we find cell types [5] and detect rare cell populations [6], but also identify differentially expressed genes and construct differentiation trajectory [7].

However, opportunities and challenges coexist. Due to plenty of genes assayed and major of them not being portrayed in scRNA-seq data, distances between data points (i.e., cells) become similar, which leads to small differences in the distances between cells and unreliable consequences in downstream analysis, such as the identification of cell subpopulations [8]. Thus, it is essential to apply dimensionality reduction methods which can learn the representations of data in a low-dimensional space. Principal component analysis (PCA) [9] is one of typical methods, which can find a set of principal components to explain the most variance of the data, and many tools such as Seurat [10] use variants of it. In addition, non-negative matrix factorization (NMF) [11] has shown its ability to identify interpretable factors from high-dimensional and sparse datasets under the constraint of no negative element. These two belong to linear methods that are insufficient in scRNA-seq data with high noise and complex structures. Deep learning could alleviate these difficulties since it derives from non-linear mapping functions. Recently many deep learning methods have been developed such as single-cell Variational Inference (scVI) [12], which design a generative model aiming at the distribution characteristics of scRNA-seq data.

After dimensionality reduction, the next step is to identify and annotate cell populations, which is also a pre-step of differential expression analysis. In most cases, clustering algorithms are employed to identify distinct groups of cells, such as K-means, hierarchical clustering, community-detection-based algorithms [13]. This strategy could detect new cell subpopulations. Alternatively, some supervised methods are developed to annotate cell subpopulations. Since these models are trained based on scRNA-seq data with cell type labels, biological information implied in these training data could be transferred to target datasets to improve the accuracy of cell type annotation. Though this strategy cannot identify new cell type, with the emergence of single cell atlas [14], tsunamic data with cell type labels could definitely provide much prior information which is valuable for the identification of cell subpopulations.

**Fig. 1:**
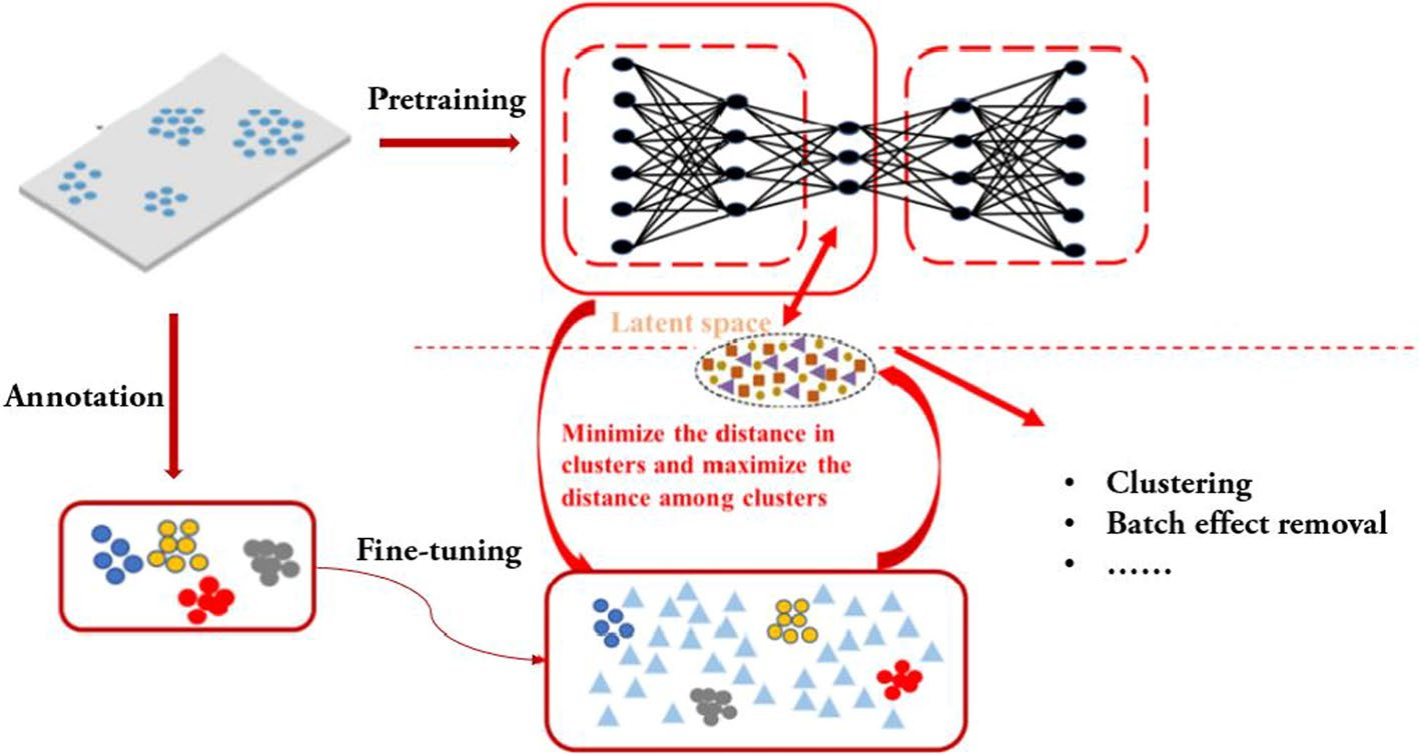
Framework of scSemiAE. (1) Annotation: predicting cell type via a classifier and labeling partial cells with high confidence; (2) Pretraining: training an autoencoder with all cells; (3) Fine-tuning: adjusting the weights of encoder using labeled cells

In this paper, we present a semi-supervised dimensionality reduction approach named scSemiAE aiming at the identification of cell subpopulations for scRNA-seq data analysis, which leverage partial cells with labels to guide the learning of an autoencoder for the target datasets (The framework of scSemiAE is shown in Fig. 1). It first employs a classifier trained from scRNA-seq data with known cell type labels to annotate cell types for target datasets and selects predictions being true with high probability, and then learns low-dimensional representations of target datasets guided by partial cells with predicted cell types. The partially labeled cells could transfer the information from other available datasets, guide to learn efficient coding of target datasets, and consequently improve the accuracy of downstream analysis. To our knowledge, netAE [15] is a semi-supervised method which uses a softmax layer to calculate classification loss. Moreover, scANVI [16] is an extension of scVI which adjusts model distribution to amplify label signals after training a basic model. We choose them as two of the baseline models.

In our experiments, we compare the latent space learned by scSemiAE with the spaces learned by netAE, scANVI, autoencoder (AE), scVI as well as principal components got from PCA in both classification and clustering tasks. When the proportion of labeled cells or the number of labeled cell subpopulations varies, the latent space learned by scSemiAE performs outstandingly in most cases, which implies that it can simplify further analysis. In addition, we show its ability to remove batch effects, which makes it more applicable in scRNA-seq data analysis.

## Results

To evaluate scSemiAE comprehensively, we implement several experiments on real datasets and compare it with both semi-supervised methods and unsupervised methods. We test the performance of scSemiAE under three scenarios, the different proportions of labeled cells, the different numbers of labeled cell subpopulations and batch effects existing. Since the datasets in these experiments must be labeled with true cell subpopulations in advance to give a standard evaluation criterion, we omit the step of cell annotation, but in real applications, cell annotation may be necessary to get partial labels with high confidence.

The methods in comparison include PCA, semi-supervised methods netAE and scANVI as well as unsupervised methods AE and scVI. Among them, AE refers to the pretraining model of scSemiAE. For fairness, we use similar network structure for all deep models in comparison. Encoder and decoder are both with two fully connected layers and the dimension of latent space is 50. The training epoch of all deep models is set to 50. Besides, to reduce the effect on performance caused by parameter regulation, these methods in comparison are tested under their default parameters and algorithm procedures. Consequently, we do not tune the hyperparameters of scSemiAE separately for each dataset but use the default settings.

Since scSemiAE can be treated as a dimensionality reduction algorithm, to show the performance, we select two representative metrics from classification field and clustering field respectively. (1) Accuracy (ACC): This one is mainly to evaluate the performance of classification. We select the k-nearest neighbor (kNN) classifier with k = 10, which is one of the simplest models for classification. For deep models, scSemiAE, AE, scVI, scANVI and netAE, embedding vectors mapping from labeled cells are used to train a kNN classifier and prediction accuracy is calculated on the unlabeled set. For PCA method, kNN is trained on principal components [15]. (2) Adjusted Rand Index (ARI) [17]: This one is mainly to evaluate the performance of clustering. We select Louvain and K-means that are two most popular clustering algorithms. They are used to group only unlabeled cells on a low-dimensional space directly and ARI is also calculated on the unlabeled cells. Louvain will stop at the best split. The size of groups, k in K-means algorithm will be set to the exact number of cell subpopulations. Besides, Uniform Manifold Approximation and Projection (UMAP) [18] are employed to give visualizations by projecting embedding into two dimensions.

### scSemiAE performs best for tests with different proportions of labeled cells

**Fig. 2:**
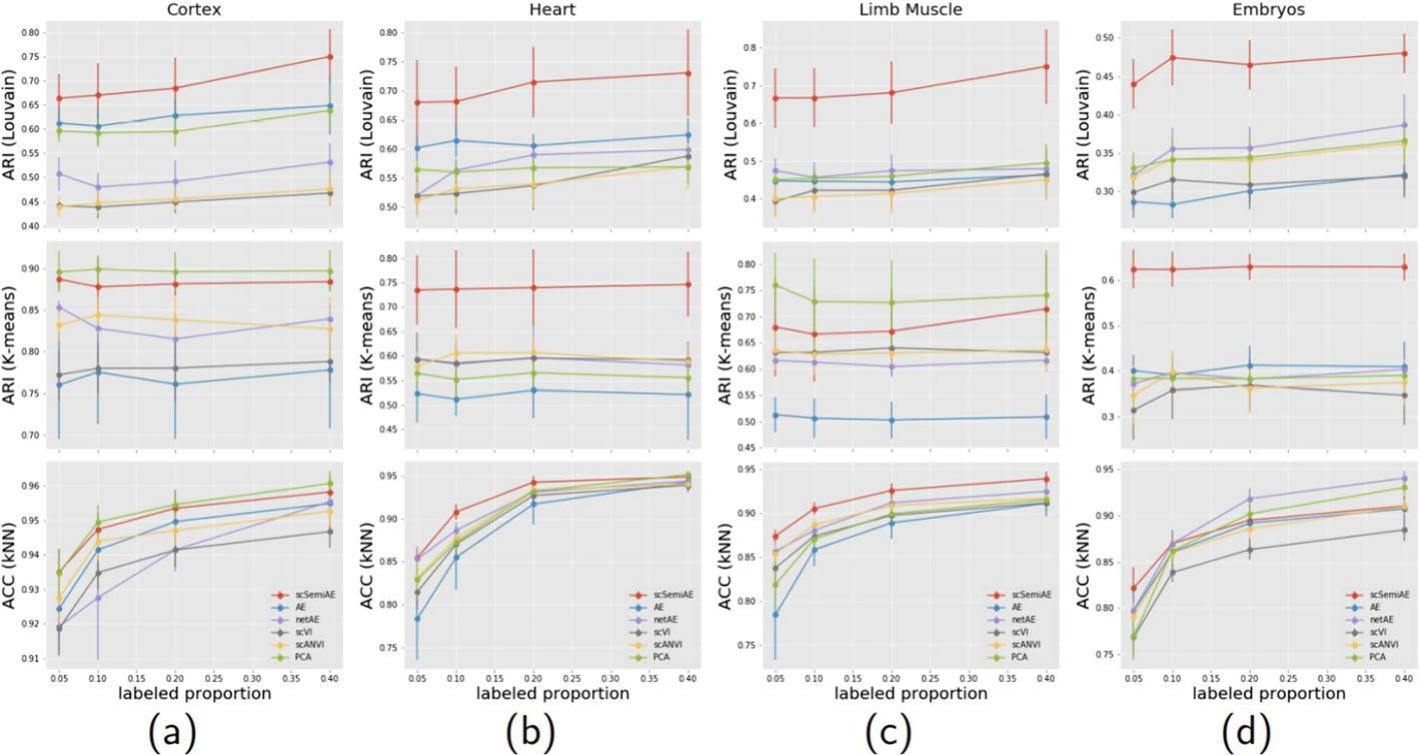
Change of ARI (Louvain & K-means) and ACC (kNN) values with the increasing labeled proportion for six methods. **a** On Cortex dataset; **b** on Heart dataset; **c** on Limb Muscle dataset; **d** on Embryos dataset

The first experiment is to test the performance of scSemiAE when the proportion of labeled cells is various. We set the proportion of labeled cells to 0.05, 0.1, 0.2, 0.4 respectively. In Fig. 2, we present the results for the first four datasets of all methods in comparison, where all the mean and standard deviation of ARI and ACC values are counted from 20 randomly sampling of labeled cells (its corresponding numerical values being shown in Additional file 1).

As shown in Fig. 2, it is very clear that scSemiAE performs best. In most tests, scSemiAE achieves the best score and especially the ARI values calculated from Louvain, scSemiAE exceeds other algorithms by 10–30%. For K-means, scSemiAE is the most stable method on multiple datasets. Though it is the second best on Cortex and Limbs Muscle datasets, scSemiAE performs much better than others in Heart and Embryos datasets. PCA fluctuates a lot and all the other models are worse than scSemiAE on ARI values. Naturally, scSemiAE should be a better choice for clustering of scRNA-seq data. Besides, the performance of scSemiAE is far beyond it of AE which is the pretraining part of scSemiAE and it demonstrates that the fine-tuning part of scSemiAE does help great. By the way, though K-means seemly performs better than Louvain, we cannot come to the conclusion quickly. Usually, the true size of cell subpopulations cannot be known beforehand and it is an important parameter for K-means. In fact, in above experiments, Louvain likely pops out more clusters than K-means, in which k is exactly set to the true size of cell subpopulations. It can explain why the ARI of Louvain looks not as good as it of K-means.

As for ACC indicator, scSemiAE achieves the best score on two datasets, Limb Muscle and Heart, and on the other two datasets, scSemiAE is competitive. What’s more, when the proportion of labeled cells is very low, such as 0.05, scSemiAE is the best one.

A series of experiments illustrate that scSemiAE should be the first choice among the semi-supervised methods and unsupervised methods in comparison since it performs best or secondly best in tests with different proportions of labeled cells.

#### scSemiAE performs best for tests with the different number of labeled cell subpopulations

In reality, there must be some cell types, especially rare cell types, which cannot be annotated by a cell type predictor since these cell types may not be detected before. Therefore, we explore the performance of scSemiAE when the number of annotated cell types is limited. In this section, due to the size of cell subpopulations in the dataset, the number of labeled cell subpopulations ranges from 2 to 5, 6 or 7. Up to $10\%$ cells of a cell subpopulation with more than 50 cells may be labeled. Three semi-supervised methods, scSemiAE, netAE and scANVI are compared.

**Fig. 3:**
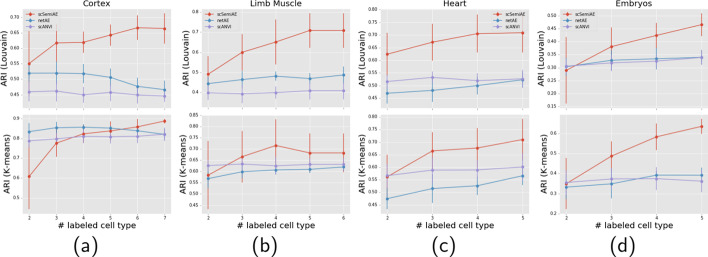
Change of ARI (Louvain & K-means) values when increasing the number of labeled cell subpopulations for three semi-supervised methods. **a** on Cortex dataset; **b** on Heart dataset; **c** on Limb Muscle dataset; **d** on Embryos dataset

The experimental results are shown in Fig. 3 (its corresponding numerical values being shown in Additional file 2). Obviously in most cases scSemiAE performs best regardless of the number of annotated cell subpopulations. From the results of Louvain algorithm, when the number of annotated cell populations is more than 2, ARI of scSemiAE achieves about 20–30% better than it of the other two methods. When the number of annotated cell populations is 2, scSemiAE outperforms much in Cortex, Heart, and Limb muscle datasets, and performs comparably in Embryos datasets. The observation from K-means almost agrees it from Louvain, scSemiAE performs best in whole, except on Cortex dataset, when the number of cell types is less than 4, netAE and scANVI outperformed scSemiAE a little.

What’s more, among three methods, scSemiAE is the only one that with the increasing of annotated cell subpopulations, the performance of clustering unlabeled cells become better. While, netAE and scANVI cannot take full advantage of annotated cells since their performances keep very stable with the increasing of annotated cell subpopulations.

As shown in Table 1, some datasets are seriously unbalanced, such as Heart datasets and among cell subpopulations of it, the most and least cells differ by two orders of magnitude. In this case, it is truly hard for clustering algorithms to simultaneously identify both common cell type and rare cell type. scSemiAE alleviates the problem because it gives better low-dimensional representations in which common cell subpopulations and rare cell subpopulations are easy to be grouped respectively. ARIs from scSemiAE for Heart datasets are much better than its from netAE and scANVI, the other two semi-supervised methods, as shown in Fig. 3.

### scSemiAE could remove batch effects

Though scSemiAE takes no extra step to remove batch effects, it does have this function since one goal of it is to make cells of the same cell type close.

**Fig. 4:**
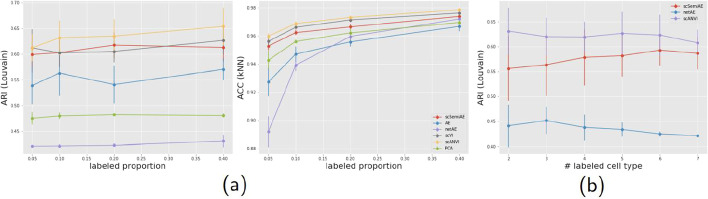
**a** Change of ARI (Louvain) and ACC (kNN) values with the increasing labeled proportion for six methods on Pancreas dataset; **b** Change of ARI (Louvain) values when increasing the number of labeled cell subpopulations for three semi-supervised methods on Pancreas dataset

The Pancreas dataset is from four different batches. We also implement the experiments mentioned above on this dataset. Among the methods, scANVI inherits from scVI the special treatment for removing batch effects, while scSemiAE and other methods do not take specific solution for it. As shown in Fig. 4 (its corresponding numerical values being shown in Additional file 3), the special treatment for removing batch effects do help scANVI and scVI outperform other methods. While, scSemiAE presents similar performance as scVI in clustering, a litter worse than the semi-supervised version scANVI, and much better than netAE and other unsupervised methods. It demonstrates that even when batch effects exist, scSemiAE could give better low-dimensional representations which make the work of clustering algorithms easier.

**Fig. 5:**
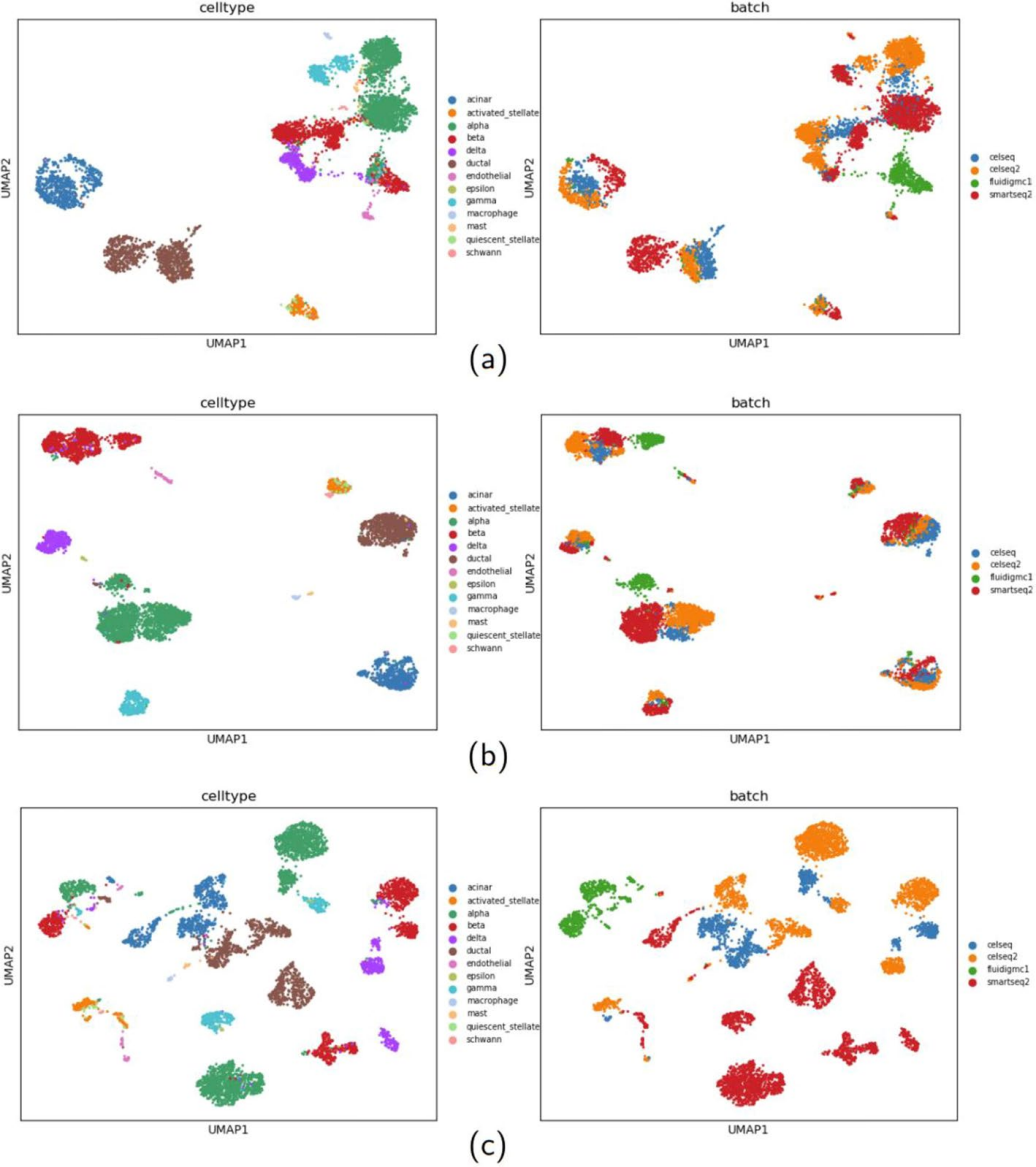
Comparison on the Pancreas dataset. **a** Visualization for the raw data; **b** Visualization for the embedding of scSemiAE; **c** Visualization for the embedding of netAE

For the Pancreas dataset, we further give visualizations of the raw data, the embedded data from scSemiAE and netAE by UMAP, shown in Fig. 5 and here the labeled proportion is set to 0.1. It is obviously shown in Fig. 5a which is from raw data that when batch effects exist, cells such as alpha cells from CelSeq2 and Fluidigm C1 are rather far away, and even worse, alpha cells of Fluidigm C1 and beta cells of Fluidigm C1 are mixed together. It illustrates that here technical variations are bigger than biological variations, while most methods for batch effects removal, such as Seurat [10], Harmony [19], LIGER [20], work under the contrary assumption. In Fig. 5b, it is very clear that scSemiAE mixes cells of the same cell subpopulation from different batches very well, and each cell subpopulation is rather separate to ease clustering. In Fig. 5c, netAE looks unable to remove batch effects, such as alpha cells of Fluidigm C1, CelSeq2 and SMART-Seq2 are very far away, so is beta cells of Fluidigm C1, CelSeq2 and SMART-Seq2. What’s worse, alpha cells and beta cells of Fluidigm C1 are much closer, just like the Fig. 5a.

The fine-tuning step of scSemiAE is to make cells of the same subpopulation close and cells among different subpopulations far away, which in some extent helps to remove batch effects even the step is guided by a few labeled cells. While netAE cannot deal with batch effects since one important part of its optimization goals is to make classification work, a classifier may find a cutline to classify different cell subpopulations while it is hard for unsupervised methods to group cells.

### scSemiAE could preserve the cell differentiation structure

**Fig. 6:**
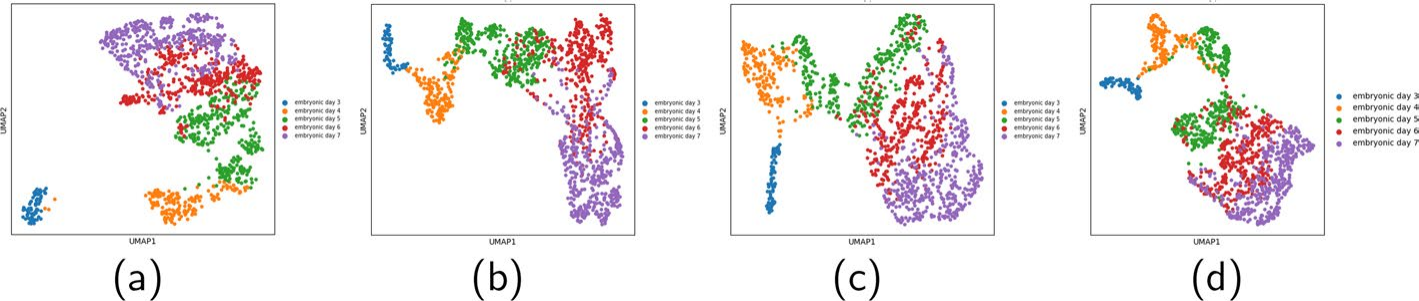
Comparison on the Embryos dataset. **a** Visualization for the raw data; **b** Visualization for the embedding of scSemiAE; **c** Visualization for the embedding of netAE; **d** Visualization for the embedding of scANVI

Embryos dataset includes 5 states of embryo development from the third day to the 7th day. UMAP visualizations for this dataset of the original space as well as the embedded spaces by scSemiAE, netAE and scANVI are shown in Fig. 6. The labeled proportion is also set to 0.1. From Fig. 6, we can figure out scSemiAE preserves the structure of the differential process of cells. It suggests that scSemiAE could provide better low-dimensional representations which could ease clustering and downstream trajectory inference.

## Discussion

Clustering is the basis of differentially expressed genes detecting and trajectory analysis. scSemiAE could help clustering algorithms find groups with more homogeny, and could preserve the cell differentiation structure, therefore it could give better support for differentially expressed genes detecting and trajectory analysis. In the future, we will consider how to emphasize the representing and detecting of rare cell types. scSemiAE could separate rare cell types from common cell types in latent space by now. However, if multiple rare cell types exist, though they could be separated, it is still hard for clustering since the too small cell subpopulations are tended to be grouped with other cell types. Our experiments show cell types with tens of cell could be separated by scSemiAE and Louvain, while if cells of a cell type is about 10, sometimes it is merged with other cell types.

## Conclusion

Due to high dimensions and high sparsity of scRNA-seq data, dimensionality reduction is an indispensable step before identifying cell subpopulations. In scRNA-seq data analysis, they are often treated as two isolated steps and the new algorithms have been introduced constantly and separately. If the dimensionality reduction algorithm can take a long view, the features we gain will benefit the identification of cell subpopulations.

By now, with the scRNA-seq data tsunami emerging, biological prior knowledge such as some common cell profiles could easily be gained and help the analysis of new datasets. In this paper, we propose scSemiAE, a semi-supervised deep learning framework combined with the thought of classification and clustering. On the one hand, we directly leverage a classifier to predict target datasets, which can provide prior knowledge; on the other hand, we encourage the low-dimensional representations of cells from the same cell subpopulation to be similar, while the representations of cells among different cell subpopulations are dissimilar. scSemiAE adopts a three-step strategy including annotation, pretraining, and fine-tuning. A series of experiments for the different proportions of labeled cells or the different numbers of labeled cell subpopulations show that the performances of scSemiAE are rather excellent. It could leverage the prior information such as some, even few labeled cells to guide the gathering of the same cell subpopulation, which could ease clustering. A byproduct of scSemiAE is that it could remove batch effects to some extent since it encourages the same cell type from different batches to gather together, which breaks the constraint that biological variations of different cell types should be greater than technical variations from different batches. In addition, it is also helpful to preserve the cell differentiation structure.

Active learning [21] is a form of semi-supervised learning, which is important techniques when labeled data are scarce. It is worth trying to integrate active learning strategy into scSemiAE to make improvement on learning efficiency and accuracy.

## Methods

The core of scSemiAE is a semi-supervised version of the traditional autoencoder. Its primary objective is to learn a nonlinear mapping function by using the prior information contained in some labeled cells so as to project high-dimensional expression of all cells to vectors in a low-dimensional space where similar cells are close and dissimilar cells are far away. In this section, we present the method of data preprocessing and the framework of scSemiAE.

### Data preprocessing

As for data preprocessing, we first filter out genes that are not expressed in any sample and cells in which any reads are not captured. Then, we normalize the raw count matrix by using Scanpy package and making the target sum as the median of total counts for cells before normalization and take the log of it. Moreover, we select the top 5000 genes with high variance, which can save training costs to some extent.

### Framework

To be more specific, the framework of scSemiAE consists of three stages: first, annotating labels for all cells using a classifier tool and picking out some of them whose labels are more likely to be right as the provider of prior information; second, pretraining with all cells on an autoencoder so that the latent characters can acquire as much effective information as possible; and third, adjusting the embedding function using labeled cells to accomplish the goal of grouping the cells with the same label and separating the cells with different labels.

In the first step, totally unannotated datasets will be annotated by existing tools, for example, SciBet [22], ScMap [23], Garnett [24], CellAssign [25], et al., which are classifiers trained from datasets with cell labels. Most classifiers output the probability that a cell belongs to a certain cell type. The label with the maximum probability is regarded as the cell annotation result. For the prediction results are not completely correct, we set a threshold to filter out cells whose predictions are without high probability to ensure the selected labels as accurate as possible. Otherwise, the cell type is labeled as unknown. As for partially unannotated datasets, we can directly start with the second step.

In the second step of pretraining, an autoencoder is trained on all preprocessed data and we can get an embedding function that output latent vectors that are compressed representations while keeping the most important information of input data.

An autoencoder is a kind of artificial neural network used to learn efficient coding of unlabeled data by training to refine nonlinear features and ignore insignificant information. We take preprocessed gene expression profiles $X=\{x_j \in R^G \}_{j=1}^{N}$ as autoencoder’s inputs, where *N* denotes the number of cells and *G* denotes the number of genes. Autoencoder usually consists of two main parts to regenerate the inputs: an encoder part that maps the inputs to a lower-dimensional embedding $Z=\{z_j \in R^D \}_{j=1}^{N}$, where *D* denotes the dimension of the latent space; a decoder part that can reconstruct the inputs as outputs ${\hat{X}}=\{{\hat{x}}_j \in R^G \}_{j=1}^{N}$ in the original space from the lower-dimensional embedding. The main idea of an autoencoder is to minimize the difference between the inputs and the outputs through optimizing the weights of the network. The loss function of the pretraining model is as follows and we choose mean squared error as data reconstruction error.

1 $$\begin{aligned} L_p = \min L(X, {\hat{X}}) = \min \left\| X - {\hat{X}} \right\| ^2 \end{aligned}$$

The third step is fine-tuning, which is a process of updating the weights of the encoder network based on partially labeled cells and finding a mapping function that can make the learned low-dimensional representations easily distinguish cells being the same cell subpopulation or not.

Let *S* be a scRNA-seq dataset. We assume that through the annotating process, *S* get some labels. *S* consists of two parts: one is gene expression matrix of labeled cells $X_L=\{x_j \in R^G \}_{j=1}^{N_L}$ and a label vector of these cells $Y_L=\{y_j \in \{1,\ldots ,K\}\}_{j=1}^{N_L}$, in which $N_L$ denotes the number of labeled cells and *K* represents the number of cell subpopulations in the labeled set; the other is gene expression matrix of unlabeled cells $X_U=\{x_j \in R^G \}_{j=1}^{N_U}$ and $N_U$ denotes the number of unlabeled cells. Of note, we assume that cell subpopulation *i* has $N_i$ cells in labeled set.

Intuitively, in the data space, cells of the same subpopulation should be close, and cells among different subpopulations should be separated. Therefore, we design the optimization goal of fine-tuning process is to learn a nonlinear mapping function $f_\theta :R^G \rightarrow R^D$ in which $\theta$ is the whole parameters of the encoder network and through the mapping of $f_\theta$, labeled cells within the same cell subpopulation will be closer and vice versa.

On the one side, average distance within each cell subpopulation $L_1$ is defined in formula (2), in which, $c_i$ means center of cell subpopulation *i* shown in formula (3) and $C_L=\{c_i\in R^D \}_{i=1}^{K}$ represents all centers in embedding space [26]. *d* is distance function and we choose squared Euclidean distance. By minimizing the $L_1$ we can get similar representations of cells within a cell subpopulation in the latent space.

2 $$\begin{aligned} L_1 = \frac{1}{K} \sum _{i=1}^{K} \frac{1}{N_i} \sum _{j=1}^{N_i} d(f_\theta (x_j), c_i) \end{aligned}$$

3 $$\begin{aligned} c_i = \frac{1}{N_i} \sum _{j=1}^{N_i} f_\theta (x_j) \end{aligned}$$

On the other side, for cells among different cell subpopulations, they deserve representations as distinct as possible. We define the distance among several cell subpopulations, $L_2$, as the minimum distance of any two centers, shown in formula (4).

4 $$\begin{aligned} L_2 = \min _{0< i_1 < i_2 \le K} d(c_{i_1}, c_{i_2}) \end{aligned}$$

The final objective function in formula (5) optimizes for the $L_1$ and $L_2$ distance jointly:

5 $$\begin{aligned} L_m = \min _{\theta } (L_1 - \lambda \times L_2) \end{aligned}$$

where $\lambda$ is a regularization constant which can balance between $L_1$ minimization and $L_2$ maximization. Through optimizing for $f_\theta$, we can obtain the latent space where we can distinguish or cluster cells easily even for unlabeled cells.

### Training strategy

In this subsection, we give our whole training strategy and default parameters settings of scSemiAE.

In the phase of pretraining, we first train $L_p$ 50 epochs on all preprocessed data with a deep autoencoder as mentioned above. Then, scSemiAE removes the decoder of the pretraining model and uses learned weights of the encoder to initialize the neural network, which can provide a prior parameter space. In the process of fine-tuning, the new loss function $L_m$ is used to re-train the encoder network for 60 epochs, in order to further obtain biological characters and preserve biological structural relationships in the latent space.

As for the details of the implementation of scSemiAE, we use two fully connected layers in the encoder and decoder respectively and the encoder has the structure, $dim_{5000} \rightarrow dim_{500} \rightarrow dim_{50}$, while the decoder has a mirror-image. For both pretraining and the beginning of fine-tuning, we use the Adam [27] optimizer with learning rate 0.001 and for fine-tuning, we adjusted the learning rate to half of the original value after every 5 epochs. What’s more, we choose layer normalization for the hidden layer and ELU function, $ELU(x) = \max (0,x) + \min (0, \alpha (exp(x) - 1)) (\alpha = 1)$, as a nonlinear activation function. For the hyperparameter, we set $\lambda = 1$, which is the regularizer between $L_1$ and $L_2$, distance within a cell subpopulation and distance among different subpopulations.

### Availability of data and materials

To test scSemiAE and other existing methods, we select five publicly annotated scRNA-Seq datasets listed as follows. More details are in Table 1.

The Cortex dataset (GSE60361) [28] concentrates on mouse brain cortex cells and contains 3005 cells with 7 cell types, and it is relatively balanced from the aspect of quantity.

Tabula Muris Senis dataset [29] is a comprehensive compendium of single-cell transcriptomic data and is available at: https://figshare.com/projects/Tabula_Muris_Se-nis/64982/, from which, we choose cells in heart and limb muscle from 18-month-old mice. The Heart and Limb Muscle datasets has 11 and 6 cell types respectively and the number of cells whithin each cell types is unbalanced, as shown in Table 1.

**Table 1:** The details for all used datasets

Dataset	# Cells	#Cell subpopulations	# Genes	# Cells of each subpopulation
Cortex	3005	7	19972	939, 820, 399, 290, 235, 224, 98
Heart	4433	11	23341	775, 458, 344, 127, 100, 93, 58, 47, 41, 8
Limb Muscle	1521	6	23341	683, 354, 205, 172, 70, 37
Embryos	1529	5	24557	466, 415, 377, 190, 81
Pancreas	6321	13	34363	2281, 1172, 1065, 711, 405, 359, 180, 61, 24, 20, 17, 14, 12

The Embryo dataset (E-MTAB-3929) [30] contains 1529 cells from human embryos with 5 cell states, corresponding to day 3 to day 7 of embryo development.

The Pancreatic dataset [31, 32] consists of 6321 human pancreatic islet cells with 34363 genes in 13 cell types sequenced by four distinct sequencing technologies, CelSeq (GSE81076), CelSeq2 (GSE85241), Fluidigm C1 (GSE86469) and SMART-Seq2 (E-MTAB-5061).

## Supplementary Information

**Additional file 1**. Results for the first experiment Change of ARI (Louvain & K-means) and ACC (kNN) values with the increasing labeled proportion for six　methods　(AE, netAE, PCA, scSemiAE, scANVI and scVI) and four datasets(Cortex, Heart, Limb Muscle and　Embryos). In addition, mean and std is the mean and standard deviation.

**Additional file 2**. Results for the second experiment Change of ARI (Louvain & K-means) values when increasing the number of labeled cell subpopulations for three　semi-supervised methods　(netAE, scSemiAE and scANVI) and four datasets(Cortex, Heart, Limb Muscle and　Embryos).

**Additional file 3**. Results for the third experiment Change of ARI (Louvain) and ACC (kNN) values with the increasing labeled proportion and change of ARI　(Louvain) values when increasing the number of labeled cell subpopulations for the Pancreas dataset.

## Data Availability

scSemiAE is available and open source at https://github.com/PlusoneD/scSemiAE, the datasets we used are listed in above and are available.

## Abbreviations

scRNA-seq: Single-cell RNA sequencing
PCA: Principal component analysis
NMF: Non-negative matrix factorization
scVI: Single-cell Variational Inference
AE: Autoencoder
ACC: Accuracy
kNN: k-Nearest neighbor
ARI: Adjusted rand index
UMAP: Uniform Manifold Approximation and Projection
